# Bronchial epithelial spheroids: an alternative culture model to investigate epithelium inflammation-mediated COPD

**DOI:** 10.1186/1465-9921-8-86

**Published:** 2007-11-26

**Authors:** Gaetan Deslee, Sandra Dury, Jeanne M Perotin, Denise Al Alam, Fabien Vitry, Rachel Boxio, Sophie C Gangloff, Moncef Guenounou, François Lebargy, Abderrazzaq Belaaouaj

**Affiliations:** 1 Service des Maladies Respiratoires, Hôpital Maison Blanche, CHU de REIMS, France; 2 INSERM UMR514, IFR 53, Hôpital Maison Blanche, CHU de REIMS, France; 3 Laboratoire d'Immuno-Pharmacologie Cellulaire et Moléculaire, EA3796, Université de Reims Champagne-Ardenne, IFR 53, Reims, France; 4 Unité Aide Méthodologique, Hôpital Maison Blanche, CHU de REIMS, France

## Abstract

**Background:**

Chronic obstructive pulmonary disease (COPD) is characterized by abnormal lung inflammation that exceeds the protective response. Various culture models using epithelial cell lines or primary cells have been used to investigate the contribution of bronchial epithelium in the exaggerated inflammation of COPD. However, these models do not mimic *in vivo *situations for several reasons (e.g, transformed epithelial cells, protease-mediated dissociation of primary cells, etc.). To circumvent these concerns, we developed a new epithelial cell culture model.

**Methods:**

Using non transformed non dissociated bronchial epithelium obtained by bronchial brushings from COPD and non-COPD smokers, we developed a 3-dimensional culture model, bronchial epithelial spheroids (BES). BES were analyzed by videomicroscopy, light microscopy, immunofluorescence, and transmission electron microscopy. We also compared the inflammatory responses of COPD and non-COPD BES. In our study, we chose to stimulate BES with lipopolycaccharide (LPS) and measured the release of the pro-inflammatory mediators interleukin-8 (IL-8) and leukotriene B4 (LTB4) and the anti-inflammatory mediator prostaglandin E2 (PGE2).

**Results:**

BES obtained from both COPD and non-COPD patients were characterized by a polarized bronchial epithelium with tight junctions and ciliary beating, composed of basal cells, secretory cells and ciliated cells. The ciliary beat frequency of ciliated cells was not significantly different between the two groups. Of interest, BES retained their characteristic features in culture up to 8 days. BES released the inflammatory mediators IL-8, PGE2 and LTB4 constitutively and following exposure to LPS. Interestingly, LPS induced a higher release of IL-8, but not PGE2 and LTB4 in COPD BES (p < 0.001) which correlated with lung function changes.

**Conclusion:**

This study provides for the first time a compelling evidence that the BES model provides an unaltered bronchial surface epithelium. More importantly, BES represent an attractive culture model to investigate the mechanisms of injuring agents that mediate epithelial cell inflammation and its contribution to COPD pathogenesis.

## Background

Chronic obstructive pulmonary disease (COPD) is characterized by progressive limitation of expiratory airflow and is associated with chronic inflammation in response to various injuring agents [1,2]. Cigarette smoke outweighs any other etiologic factor in the development of COPD. And exacerbations mediated for instance by respiratory infections have a direct effect on the disease worsening and acceleration of lung function loss [3]. COPD is recognized as a major health problem worldwide resulting in large consumption of health care resources [4]. Remarkably, advances in therapy against COPD are still limited due in part to poor understanding of the mechanisms underlying the setting and/or progression of this disease.

Among the hallmarks of COPD are chronic inflammation, injury of both parenchyma and epithelial lining, and recruitment/activation of inflammatory cells (neutrophils, macrophages and CD8+ T cells) triggered in part by mediators derived from the epithelium [5-8]. In controlled situations, the bronchial epithelium represents the first line of defense and protects the lung by acting as a physicochemical barrier of the submucosa. This tissue is also able to mount an inflammatory response releasing mediators following exposure to insulting agents including cigarette smoke [9], cytokines [10-13], and infectious pathogens or their products such as lipopolysaccharide (LPS) [14-16]. However, in the setting of overwhelming conditions such as in COPD, there appears to be an abnormal inflammatory response in the lungs beyond the normal protective response. But, the mechanisms of airway epithelium inflammation and their contribution to COPD development are not entirely clear.

Different systems have been developed to investigate the role of airway epithelium in COPD. Morphologic analyses using surgical specimens or bronchial biopsies from COPD and non-COPD smokers demonstrated an enhanced inflammatory cell infiltration in COPD, goblet cell hyperplasia and plugging associated with mucus hypersecretion in both groups [17]. Of note, these studies did not reveal discernable histologic differences in bronchial surface epithelium between the two groups. Functional analyses or response studies to stimuli could not be carried out using these tissues. A number of cell culture models were established to study the role of the epithelium in COPD ranging from its response to insults, differentiation, injury, and regeneration. These models include cell culture on uncoated or coated wells, air-liquid interface system, and xenograft model. Application of either model to epithelial cell lines or primary epithelial cells provides undoubtedly insights in the biology of epithelium. But, extrapolation of data obtained from these studies to *in vivo *situations could be misleading due to a number of concerns. For example, epithelial cell lines employed in most of the studies are transformed cells. Primary bronchial epithelial cells, when removed from their host tissue, are dissociated (e.g., trypsinisation). And when grown as monolayers, they undergo dedifferentiation, proliferation and loose some of their specific functions. Indeed, epithelial cells dissociated from nasal polyps dedifferentiate rapidly with loss of ciliated cells and disappearance of tight junctions [18]. More recently, we and others have developed three-dimensional (3-D) cultures of human epithelial cells, spheroids, using lung epithelial cell lines or cells derived from nasal epithelium [19-24]. Compared to 2-D models, the 3-D culture model keeps the airway epithelium in a well-differentiated and polarized state, as demonstrated by an enhanced expression of functional tight junctions proteins, an increase in expression of cell-specific markers and a greater induction in proinflammatory cytokines following stimulation [19]. Although, these studies suggest 3-D cultures as a more physiologically relevant model to examine airway epithelium functions, 3-D culture from nasal epithelium, a widely used model, may not represent *in vivo *situations due to cell changes resulting from protease-induced dissociation, proliferation, secondary aggregation and differentiation.

Our goals in the current study were two fold. First, we sought to develop for the first time a bronchial epithelial spheroids (BES) model, a 3-D culture system, using non transformed non dissociated bronchial brushings obtained from COPD and non-COPD patients. Second, we validated this model by comparing the responses of both types of BES to lipopolysaccharide (LPS), a ubiquitous endotoxin. Our findings show that COPD and non-COPD smokers-derived brushings form spontaneously 3-D BES. Both types of BES are characterized by a polarized bronchial epithelium with tight cell-cell junctions, composed of basal cells, secretory cells and ciliated cells. We also provide evidence that COPD, but not non-COPD BES, exhibit an enhanced inflammatory response to LPS.

## Methods

### Patients

Patients referred to Reims University Hospital to undergo flexible bronchoscopy were screened for inclusion in the present study. All the patients were current or ex-smokers (>10 pack-years). The selection of COPD patients was established on the basis of the Global Initiative for Chronic Obstructive Lung Disease guidelines [25] as a ratio of FEV_1_/FVC less than 0.7 after the administration of 200 μg of salbutamol. Patients were excluded from the study when one of the following conditions was present : recent history of exacerbation and/or respiratory tract infection, an increase in FEV_1 _> 20%/baseline or >200 mL at 30 min following 200 μg of inhaled salbutamol, history of asthma, and use of inhaled or systemic corticosteroids. Patients were asked not to smoke 12 hours before bronchoscopy. Detailed informations about the patients included their smoking habits (current smoking status and number of pack-years), drug history, age, weight and height.

Lung function was determined for all patients, including FEV_1_, FVC, FEV_1_/FVC, reversibility of airflow limitation (ΔFEV_1_) measured after administration of 200 μg salbutamol, total lung capacity (TLC), residual volume (RV), and diffusing capacity for carbon monoxide per liter alveolar volume (K_CO_) (BodyBox 5500, Medisoft, Sorinnes, Belgium). For all COPD patients, the BODE index was computed from body-mass index, degree of airflow obstruction, dyspnea score (Modified Medical Research Council scale), and exercise capacity measured by the six-minute-walk test [26].

The study was approved by the Consultative Committee Protecting Persons in Biomedical Research (CCPPRB) of Champagne-Ardenne. All the subjects gave their informed written consent.

### Fiberoptic bronchoscopy and bronchial brushings

After local anesthesia with 2% lidocaine, a fiberoptic bronchoscope (Olympus, Paris, France) was inserted into the trachea and airways were systematically examined. Bronchial epithelium was obtained by gentle brushing of segmental bronchi under visual control by mean of a cytology brush (Olympus, Paris, France). Each patient underwent 6 bronchial brushings. Brushes were processed immediately to carry out our studies.

### Culture of bronchial epithelial spheroids

Samples obtained from bronchial brushings were gently centrifuged (50 g for 5 min), and resuspended in 2 mL RPMI-1640 (Invitrogen, Carlsbad, CA) supplemented with insulin (1 μg/ml; Sigma Chemical, St Louis, MO), Apo-transferrin (1 μg/ml; Serva, Heidelberg, Germany), epidermal growth factor (10 ng/ml; Sigma), hydrocortisone (0.5 μg/ml; Sigma), retinoic acid (2,5 μg/ml; Sigma), amphotericin B (2,5 μg/ml; Sigma), penicillin (200 U/ml) and streptomycin (100 U/ml). Bronchial brushings were then treated with a mucolytic agent (acetylcysteine 2.5%) (Bristol-Myers Squibb, Rueil-Malmaison, France) for 15 min. Epithelial sheets were then collected and washed two times. Pooled bronchial epithelial sheets were then resuspended in 2 ml medium supplemented with fetal calf serum 10% and cultured in 24-well flat-bottomed culture plates at 37°C, 5% CO2 for 24 h. Under static conditions, bronchial epithelial sheets formed rapidly and spontaneously distinctive bronchial epithelial spheroids (BES). BES were in suspension and did not adhere to the wells. Next, BES were gently transferred to new culture plate, washed one time and cultured for various time periods (1 to 8 days). Morphological and functional analyses were carried out at designated time points.

### Tissue section preparation and immunofluorescence

BES were gently centrifuged and cryofixed in liquid nitrogen and stored at -80°C as previously described [18]. Transverse frozen sections (5 μm thick) were placed on gelatin-coated glass slides and fixed in methanol (-20°C) for 10 min. Slides were washed twice in PBS, incubated in 1% bovine serum albumin (BSA) for 5 min and then incubated for 1 h at room temperature with the following primary antibodies: anti-Cytokeratin 13 (Sigma) anti-Cytokeratin 18 (Sigma), anti-MUC5AC (gift from JP Aubert, Lille, France), anti-occludin (Zymed, San Francisco, CA), anti-zonula occludens (ZO-1) (Zymed), anti E-cadherin (R & D Systems, Minneapolis, MN), anti-IL-8 (Biosource International, Camarillo, CA), and Ki67 antigen (MIB-1 clone) (Immunotech, Marseille, France). Preimmune sera were used as negative controls. Next, slides were washed three times and incubated with the biotinylated secondary antibody for 1 h at room temperature. After washing the slides three times, Alexa Fluor 488-conjugated streptavidin (1:100; Molecular Probes) was added. Nuclei were couterstained with Harris haematoxylein solution (Sigma), mounted in citifluor antifading solution (Agar Scientific, Essex, United Kingdom), and observed with an Axiophot microscope (Zeiss, Le Pecq, France) at a magnification of × 40.

### Transmission Electron Microscopy

BES were fixed in 2% glutaraldehyde-PBS for 1 h at room temperature and then postfixed with 1% osmium tetroxide at 4°C. BES were then dehydrated and embedded in increasing concentrations of Epon diluted in ethanol and ranging from 50% to 100%. Polymerisation for 78 h at 60°C was then carried out. Ultrathin sections (80 nm) were cut on a microtome, mounted on copper grids, and stained with uranyl acetate and lead citrate. The sections were observed on a J.E.O.L. 200 × transmission electron microscope operating at 75 kV.

### Videomicroscopy and measurement of ciliary beat frequency

BES plates were placed in a culture chamber at 37°C, 5% CO2, and was followed overtime by videomicroscopy using a CCD video-camera connected to an Axiophot microscope (Zeiss, Le Pecq, France) with ×40 objective. In order to assess the ciliary beating, the video images of active ciliated cells were displayed on a video screen. A photodetector placed on a ciliated cell on the video detects an analogic signal which was filtered, amplified and numerized to obtain the ciliary beating frequency, as previously described [27]. Ciliary beat frequency was measured on five different BES per preparation, and the mean ciliary beat frequency was determined by averaging five different measures.

### LPS stimulation of BES and ELISA

For LPS dose-response experiments, BES obtained from each patient were equally divided in four different wells in a 1 ml culture medium and incubated with 0, 0.1, 1 or 10 μg/ml *Pseudomonas aeruginosa *LPS (Calbiochem, San Diego, CA) for 24 h at 37°C, 5% CO2. Next, BES were treated with 10 μg/ml LPS and supernatants collected at 1, 4, 8 and 24 h for time-course experiments.

Supernatants were separated to BES by centrifugation. IL-8, PGE2 and LTB4 levels were measured in the supernatants using quantitative sandwich immunoassay technique (R & D Systems, Minneapolis, MN) following the manufacture's instructions. The cell pellet of BES was treated with RIPA buffer (50 nM Tris [pH 7.4], 150 mM NaCl, 1% Igepal [vol/vol], 1% sodium deoxycholate [wt/vol], 5 mM iodoacetamide, 0.1% sodium dodecyl sulfate [SDS, wt/vol]) containing a protease inhibitor cocktail (Roche Diagnostics GmbH, Mannheim, Germany). BES protein concentrations were determined using BC assay protein quantification kit (Interchim, Montluçon, France). Levels of IL-8, PGE2 and LTB4 were normalized to BES total protein concentrations, and were expressed in pg.mg total protein^-1^. LPS-induced fold-increase of the inflammatory mediators were determined as ratio of LPS-induced and basal levels. Following LPS dose-response and time course experiments, 10 μg/ml LPS and 24 h culture time were chosen as optimal conditions and were used for subsequent experiments.

Next, levels of IL-8, PGE2, and LTB4 at basal state and following LPS stimulation were assessed using BES from well-characterized 16 COPD and 13 non-COPD smokers (Table 1). BES from each patient were divided in two wells and incubated with or without 10 μg/ml LPS for 24 h. IL-8, PGE2 and LTB4 levels in supernatants were determined as described above. Correlations between levels of inflammatory mediators and the clinical/functional parameters of patients were determined.

**Table 1:** Physiological characteristics of study population.

	COPD Patients (n = 16)	Non-COPD Smokers (n = 13)	p value
Age, yr	57.5 ± 9.9	56.8 ± 11.1	NS
Sex (M/F)	13/3	11/2	NS
Weight, kg	77.1 ± 23.2	69.1 ± 15.1	NS
Height, m	1.71 ± 0.09	1.71 ± 0.10	NS
BMI, kg/m^2^	26.03 ± 6.85	23.40 ± 3.84	NS
PostBD-FEV_1_, %	55.38 ± 13.7	100.77 ± 9.64	<0.01
FVC, %	82.31 ± 12.13	104.45 ± 7.87	<0.01
Post-BD-FEV_1_/FVC, %	53.06 ± 10.41	77.08 ± 2.96	<0.01
TLC, %	110.56 ± 19.22	97.77 ± 9.42	<0.01
RV, %	155.69 ± 49.63	96.89 ± 28.01	<0.01
K_CO_, %	69.63 ± 21.07	87.30 ± 10.00	<0.01
Smoking status, Current/Former	8/8	6/7	NS
Pack years of smoking	44.44 ± 15.03	25.92 ± 9.37	<0.05
Moderate/Severe COPD	10/6	-	-
MMRC Dyspnea scale	2.13 ± 0.62	-	-
Distance walked in 6 min, m	339 ± 90	-	-
BODE Index	3.25 ± 2.66	-	-

BD = bronchodilator, BMI = body mass index, F = Female; M = Male; NS = statistically non significant. Values are presented as mean ± SD. Mann-Whitney U-test.

### Statistical analysis

Data are expressed as mean ± SD. A Mann-Whitney U-test was used to compare the groups and the correlations between variables were calculated by means of the Spearman's rank correlation test. A p < 0.05 was considered as significant. A multivariate analysis, including variables showing significance correlation to LPS-induced IL-8 in the univariate analysis, was performed with a multiple linear regression model.

## Results

### BES exhibit characteristic features of intact bronchial surface epithelium

**Figure 1:**
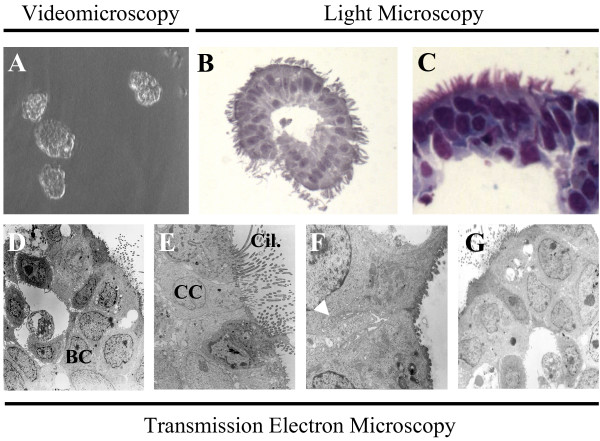
**Morphology of bronchial epithelial spheroids**. Bronchial sheets were sampled by brushings from COPD and non-COPD patients. (*A*) Videomicroscopy (see video online) shows bronchial epithelial sheets-derived spheroids. (*B,C*) Circular pseudostratified epithelium with columnar ciliated cells facing outside, and small pyramidal basal cells. Note, the presence of a central lumen. (*D,E,F*) Cohesive epithelial cells with close interdigitations and cilia facing outside. (*G*) Phenotypic and structural characteristics of spheroids were maintained up to 8 days. Micrographs are representative of spheroids obtained from COPD and non-COPD patients (n = 11). *A*, ×10; *B*, ×40,*C*, ×80; *D*, ×800; *E*, ×3000; *F*, ×5000; G, ×800. BC, basal cells; CC, ciliated cells; Cil., cilia; arrow indicates cell interdigitations.

Non dissociated epithelial sheets obtained by bronchial brushings formed rapidly and spontaneously free-floating bronchial epithelial spheroids (BES) rolling in the culture medium (Fig. 1A, videomicroscopy). This 3-dimensional structure consisted of a circular pseudostratified epithelium containing columnar cells with cilia facing outside, and small pyramidal cells with a low cytoplasmic/nuclear ratio (Fig. 1B,C,D,E). Tight cell-cell junctions and interdigitations were maintained in BES (Fig. 1F). A central lumen was present in most BES. Of interest, BES were maintained in culture up to 8 days without any noticeable cell disaggregation. Structurally, BES cultured for 1 (Fig. 1D–F) or 8 (Fig. 1G) days showed similar features.

**Figure 2:**
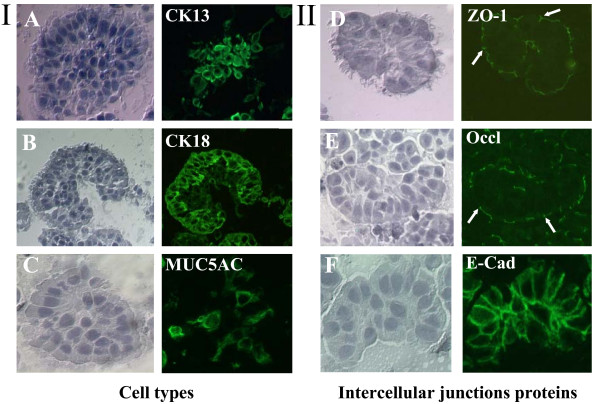
**Immunofluorescence staining of bronchial epithelium spheroids**. I and II) Right panels of immunostained BES using antibodies specific to various cells types and intercellular junction proteins. Left panels show bright field images. (*A*) Anti-cytokeratin 13 antibody (CK13) for basal cells. (*B*) Anti-cytokeratin 18 (CK18) for ciliated cells. (*C*) Anti-mucin 5AC (MUC5AC) for secreted cells. (*D,E,F*) Antibodies against intercellular junction proteins zonula occludens-1 (ZO-1), Occludin (Occl) and E-Cadherin (E-Cad). Arrows depict staining for ZO-1 and Occludin in the intercellular junctions. Micrographs are representative of spheroids from COPD and non-COPD patients (n = 7).

Immunofluorescence microscopy analyses found that COPD and non-COPD BES comprised basal cells (CK13+), ciliated cells (CK18+), and few secretory cells (MUC5AC+) (Fig. 2A,B,C). Immunofluorescence staining of ZO-1, Occludin and E-Cadherin revealed distribution of these proteins in the intercellular junctions (Fig. 2D,E,F; arrows). A non-specific patchy staining could be observed on the apical (for ZO-1 and Occludin) and basal (for E-cadherin) sides of spheroids.

No proliferation was observed as judged by the absence of immunostaining for the nuclear marker Ki67 (data not shown). BES remained tight throughout our study, as no cell detachment or lysis was detected. Cell viability of BES was >95% when evaluated by trypan blue exclusion assay.

**Figure 3:**
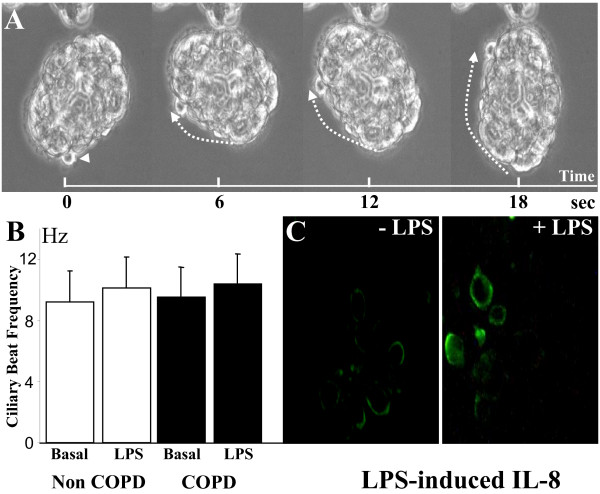
**Functional analyses of bronchial epithelial spheroids**. (*A*) Videomicroscopy shows ciliary beating associated with rolling of spheroids (see video online). (*B*). The ciliary beat frequency was similar in spheroids from both COPD and non-COPD smokers (n = 7) both at basal state (Basal) and after LPS stimulation (10 μg/ml LPS for 24 h) (LPS). (*C*) Representative micrographs of untreated (-LPS) and LPS-treated (+LPS) COPD spheroids showing LPS-enhanced expression of IL-8.

The BES rolling, as shown by videomicroscopy, was associated with ciliary beating (Fig. 3A, videomicroscopy). Interestingly, the ciliary beat frequency was not different between COPD and non-COPD BES (9.51 ± 1.34 Hz versus 9.22 ± 1.66 Hz, respectively) (Fig. 3B). Also, exposure of BES to LPS (10 μg/ml LPS for 24 h) resulted in slight but similar increase of ciliary beat frequency in both groups (Fig. 3B). Immunofluorescence staining for IL-8 found enhanced immunoreactivity following treatment of BES with LPS (Fig. 3C). All together, these data suggest that BES represent an intact bronchial epithelium that responds to stimulation.

### Enhanced release of LPS-induced IL-8, but not PGE2 and LTB4, by COPD BES

**Figure 4:**
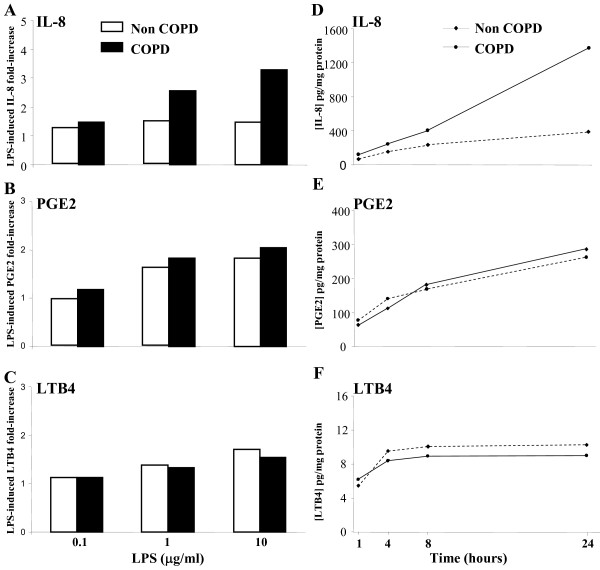
**Release of IL-8, PGE2 and LTB4 by bronchial epithelial spheroids in function of LPS dose and time**. (*A,B,C*) BES from non-COPD (open bars) and COPD smokers (filled bars) were exposed to various concentrations of LPS for 24 h. Data are expressed as fold increase of IL-8, PGE2 and LTB4 by comparison to basal levels. The findings are illustrative of 5 independent experiments. (*D,E,F*) LPS treatment (10 μg/ml) of COPD and non-COPD smokers BES for 1, 4, 8 and 24 h. Data of IL-8, PGE2 and LTB4 protein levels are expressed in pg.mg total protein^-1^. The findings are illustrative of 4 independent experiments.

Next, we determined the relevance of BES model to study the contribution of the bronchial surface epithelium to COPD airway inflammation. We obtained bronchial brushings from well-characterized COPD and non-COPD smokers. Freshly cultured spheroids were then exposed to LPS, an ubiquitous contaminant endotoxin. We incubated BES with different concentrations of LPS and examined their ability to induce the expression of the mediators IL-8, PGE2 and LTB4. Following LPS treatment for 24 h, IL-8, PGE2 and LTB4 levels increased in a LPS dose-dependent manner (0.1 to 10 μg/mL) in both COPD and non-COPD BES (Fig. 4A,B,C). These dose response and time course experiments found that 10 μg/mL LPS treatment resulted in a statistically significant increase of IL-8, but not LTB4 and PGE2, in COPD BES (p < 0.001) by comparison to non-COPD BES. Also, LPS-induced release of IL-8 and PGE2 from COPD and non-COPD BES increased progressively overtime and peaked by 24 h, whereas LTB4 release increased up to 4 h and remained constant thereafter (Fig. 4D,E,F). All the subsequent studies were carried out with 10 μg/mL LPS and 24 h culture time.

**Figure 5:**
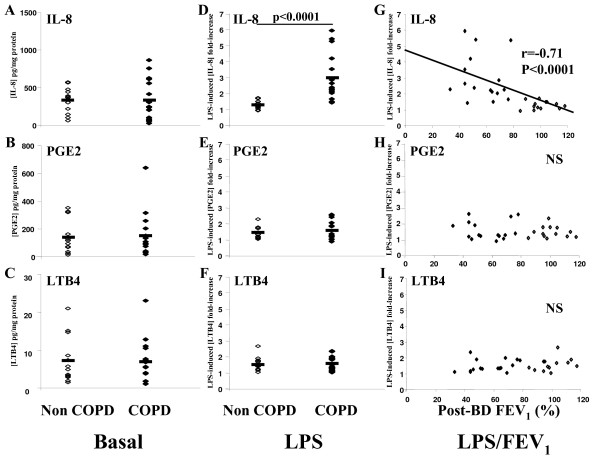
**Enhanced LPS-induced release of IL-8, but not PGE2 and LTB4 in COPD spheroids**. (*A,B,C*) Levels of IL-8, PGE2 and LTB4 in untreated spheroids. Note, no significant differences could be detected between COPD and non-COPD BES. Results are expressed as pg of mediators per mg protein of BES. (*D,E,F*) Levels of IL-8, PGE2 and LTB4 after LPS stimulation (10 μg/ml LPS for 24 h). Results are expressed as fold increase by comparison to untreated conditions. (*G,H,I*) Correlations between LPS-induced IL-8, PGE2 and LTB4 fold-increase and postbronchodilator FEV_1_. Results are obtained from 16 COPD smokers (filled rhombs) and 13 non-COPD smokers (open rhombs). Mann-Whitney U test for comparisons between groups. Correlations between variables were calculated by means of the Spearman's rank correlation test.

Next, we investigated BES obtained from larger patients groups, 16 COPD smokers and 13 non-COPD smokers (Table 1). Bronchial brushes collected from COPD and non-COPD smokers displayed the same capacity to generate BES *in vitro*. BES responses were compared in the absence or presence of LPS. Untreated COPD and non-COPD BES released the same levels of IL-8, PGE2 and LTB4 (Fig. 5A,B,C). After LPS stimulation (10 μg/mL for 24 h), there was a 3-fold increase of IL-8 in COPD BES by comparison to non-COPD BES (Fig. 5D), whereas LPS-induced PGE2 and LTB4 release were similar in both COPD and non-COPD BES (Fig. 5E,F).

### LPS-induced IL-8 release by BES correlates with clinical/functional parameters of patients

**Figure 6:**
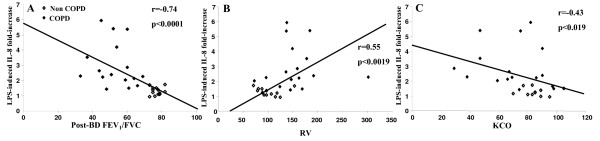
**Relationship between LPS-induced IL-8 increase and clinical/functional parameters**. (*A,B,C*) Correlations between LPS-induced IL-8 fold-increase and postbronchodilator FEV_1_/FVC ratio (Post-BD FEV_1_/FVC) (*A*), residual volume (RV) (*B*), and diffusing capacity for monoxide per liter alveolar volume (K_CO_) (*C*).

The LPS-enhanced secretion of IL-8 was inversely correlated with the level of obstruction (post-bronchodilator FEV_1_). No correlation was established between post bronchodilator FEV_1 _and PGE2 or LTB4 release (Fig. 5G,H,I). Because of the significant correlation between FEV_1 _and LPS-induced IL-8 release, other clinical and functional parameters were examined. In univariate analysis including all COPD and non-COPD smokers, basal levels of IL-8 release did not correlate with any clinical and functional parameters of the patients, and LPS-induced IL-8 release was not correlated with age, sex, weight, height and BMI (data not shown). Interestingly, a significant correlation was observed between LPS-induced IL-8 release and FEV_1_/FVC, RV and K_CO _(Fig. 6A,B,C). The smoking status (current/former) correlated with neither basal nor LPS-induced IL-8 release. In univariate analysis, the number of pack-years of smoking was correlated with LPS-induced IL-8 release (p < 0.01). Multivariate analysis demonstrated, however, that postbronchodilator FEV_1_, but not pack-years of smoking, correlated independently with LPS-induced IL-8 release (r^2 ^= 0.35, p < 0.001). No correlation was observed between basal and LPS-induced release of PGE2 and LTB4 by BES and clinical and functional parameters of COPD and non-COPD patients (data not shown).

## Discussion

In the present study, we developed for the first time a 3-D cell culture model, bronchial epithelial spheroids (BES) that should better our understanding of the role of bronchial epithelium inflammation in COPD. By comparison to other 2-D or 3-D epithelial culture systems, this model offers several advantages. The development of this culture system is less time-consuming and does not require any specific treatment. In fact, by avoiding trypsin or protease-mediated cell dissociation, bronchial cells are kept in their native intact states and do not undergo dedifferentiation and/or proliferation as occurs in most models. This "explant" type like system derived from bronchial brushings resembles a native surface bronchial epithelium. Comparative analyses found that both COPD and non-COPD bronchial epithelial sheets have the capacity to form spheroids. Morphologic analyses showed in both COPD and non-COPD BES a fully differentiated and polarized pseudostratified epithelium consisting of basal cells, ciliated cells and secretory cells. Tight junctions were maintained as judged by immunostaining for intercellular junction complexes ZO-1, occludin and E-cadherin. Of note, staining for these protein junctions on the apical and basolateral sides of spheroids has been reported in other studies using biopsies or lung specimen [28-30]. Whether this corresponds to non-specific staining or the presence of epitopes recognized by the antibodies remains to be determined.

Interestingly, BES could be maintained in culture for at least 8 days. Contrary to previous studies using nasal epithelium that showed a lower ciliary beat frequency in COPD [31], no differences were found between COPD and non-COPD BES either in the basal state or after LPS treatment. Compared to nasal epithelial cells, BES are best suited to investigate the inflammatory mechanisms of bronchial epithelium and their contribution to COPD pathogenesis. Furthermore, bronchial, but not nasal brushings, generate sufficient numbers of spheroids to carry out cellular and molecular studies.

To determine the relevance of our culture model to study COPD, we chose purposely LPS as an injuring agent and analyzed its effect on BES. We examined expression levels of the neutrophil chemoattractant pro-inflammatory mediators IL-8 and LTB4, and the anti-inflammatory mediator PGE2 [32-34] in COPD and non-COPD BES. In the absence of treatment, both COPD and non-COPD spheroids released readily and similarly detectable amounts of IL-8, PGE2, and LTB4. Our findings are in contrast with others studies, which showed changes in levels of the inflammatory mediator IL-8 [11-13]. These studies used 2-D primary epithelial cell cultures from bronchial brushings or biopsies, suggesting that differences in data could be related to cell "manipulations" (e.g., protease-induced cell dissociation, proliferation and 2-D culture). Exposure of BES to LPS resulted in an enhanced release of IL-8 in a time and LPS dose-dependant fashion and peaked by 24 h culture at 10 μg/ml LPS. Of note, the LPS concentration needed to activate spheroids was 10 to 100 fold higher than that used to stimulate monocytes/macrophages [35,36]. We found that LPS-stimulated COPD BES released higher levels of IL-8 than non-COPD BES, suggesting an enhanced epithelial inflammatory response to LPS in COPD. IL-8 is involved in the recruitment and activation of neutrophils in COPD, thereby contributing to COPD airway inflammation, particularly in the setting of infection-mediated COPD exacerbations [32,33]. Since toll-like receptor 4 (TLR-4) is crucial for effective response to LPS [37-39], studies are underway to investigate the role of this receptor in IL-8 expression in COPD.

To our knowledge no previous studies have analyzed basal or LPS-induced PGE2 and LTB4 release by bronchial epithelium in COPD. In the present work, we show that BES produce PGE2 and LTB4. However, we did not observe differences in the release of these mediators between COPD and non-COPD BES. Other studies have shown that levels of PGE2 and LTB4 are increased in exhaled breath in COPD, and LTB4 is increased in sputa and exhaled breaths in COPD exacerbations [40-43]. While the cellular sources of LTB4 and PGE2 in airways include macrophages and neutrophils, cell co-culture studies have shown that epithelial cells play a direct role in the synthesis of inflammatory cell-derived LTB4 and PGE2 [44,45]. The contribution of bronchial epithelium to expression levels of these mediators and the development of COPD remain to be defined.

To determine the clinical importance of our findings to COPD, we assessed the relationship between levels of released inflammatory mediators and clinical/functional characteristics of smokers with or without COPD. LPS-induced, but not constitutive, IL-8 release correlated with the degree of obstruction (FEV_1_, FEV_1_/FVC) and air trapping (RV), but not with the smoking status. As expected, it should be pointed that LPS-induced IL-8 levels varied among COPD patients, suggesting that epithelial cell responsiveness to LPS cannot alone explain the complex inflammatory process of COPD.

Cigarette smoke accounts for more than 90% of COPD, but only 15 to 20% of smokers develop clinically significant COPD [5,7]. Our study compared for the first time basal and LPS-induced inflammatory response of bronchial epithelium of smokers with or without COPD, and demonstrated a clear differential epithelial inflammatory response to LPS in COPD. The bacterial endotoxin LPS is an ubiquitous contaminant of environment and bioactive LPS constitutes 0.12–0.2 microgram/cigarette [46]. Experimental LPS inhalation in healthy subjects induces an increase of lung inflammatory cells and pro-inflammatory mediators, and chronic exposure to endotoxin has been shown to be associated with occupational COPD [47-50]. Hasday et al. suggested that cigarette-derived LPS contributes to COPD pathogenesis in smokers [46]. In mice, chronic lung instillation of LPS induces a COPD-like inflammation [51]. These observations together with our data suggest the importance of LPS-mediated bronchial epithelial response in the chronicity of inflammation in COPD. Our data using spheroid model provide information regarding the contribution of LPS (cigarette or bacterial infection-mediated exacerbations) to long-term decline in lung function in COPD [3].

## Conclusion

In conclusion, the spheroid culture model provides an original system that should better our understanding of COPD pathogenesis. This model should allow to investigate the role(s) of bronchial epithelial cells in COPD as well as other lung pathologies. In this culture system, not only bronchial epithelial cells are kept in a native non-transformed well-differentiated epithelium, but the cells maintain their *in vivo *morphological and functional characteristics. Thus, the mechanisms of interactions between various injuring agents/inflammatory stimuli and airway epithelium could be studied using the spheroid model in order to enhance our understanding of COPD pathogenesis and perhaps other lung inflammatory diseases, without the confounding effects frequently encountered in other tissue culture models.

## Competing interests

The author(s) declare that they have no competing interests.

## Authors' contributions

GD, FL and AB were involved in the concept and design of all experiments. GD, SD, JMP, DA, FV, RB, SCG, MG, FL, AB were involved in analysis, interpretation of data and helped to draft the manuscript. FV was involved in statistical analysis of the data. GD, FL and EB coordinated and prepared the manuscript. All authors read and approved the final manuscript.
